# In vivo alpha-V beta-3 integrin expression in human aortic atherosclerosis

**DOI:** 10.1136/heartjnl-2019-315103

**Published:** 2019-08-17

**Authors:** William S Jenkins, Alex T Vesey, Anna Vickers, Anoushka Neale, Catriona Moles, Martin Connell, Nikhil Vilas Joshi, Christophe Lucatelli, Alison M Fletcher, James C Spratt, Saeed Mirsadraee, Edwin JR van Beek, James HF Rudd, David E Newby, Marc R Dweck

**Affiliations:** 1 British Heart Foundation Centre for Cardiovascular Science, University of Edinburgh, Edinburgh, UK; 2 Clinical Research Imaging Centre, University of Edinburgh, Edinburgh, UK; 3 Division of Cardiovascular Medicine, University of Cambridge, Cambridge, UK

**Keywords:** atherosclerosis, positron emission tomography, integrin, computed tomography

## Abstract

**Objectives:**

Intraplaque angiogenesis and inflammation are key promoters of atherosclerosis and are mediated by the alpha-V beta-3 (α_v_β_3_) integrin pathway. We investigated the applicability of the α_v_β_3_-integrin receptor-selective positron emission tomography (PET) radiotracer 18F-fluciclatide in assessing human aortic atherosclerosis.

**Methods:**

Vascular 18F-fluciclatide binding was evaluated using ex vivo analysis of carotid endarterectomy samples with autoradiography and immunohistochemistry, and in vivo kinetic modelling following radiotracer administration. Forty-six subjects with a spectrum of atherosclerotic disease categorised as stable (n=27) or unstable (n=19; recent myocardial infarction) underwent PET and CT imaging of the thorax after administration of 229 (IQR 217–237) MBq 18F-fluciclatide. Thoracic aortic 18F-fluciclatide uptake was quantified on fused PET-CT images and corrected for blood-pool activity using the maximum tissue-to-background ratio (TBR_max_). Aortic atherosclerotic burden was quantified by CT wall thickness, plaque volume and calcium scoring.

**Results:**

18F-Fluciclatide uptake co-localised with regions of increased α_v_β_3_ integrin expression, and markers of inflammation and angiogenesis. 18F-Fluciclatide vascular uptake was confirmed in vivo using kinetic modelling, and on static imaging correlated with measures of aortic atherosclerotic burden: wall thickness (r=0.57, p=0.001), total plaque volume (r=0.56, p=0.001) and aortic CT calcium score (r=0.37, p=0.01). Patients with recent myocardial infarction had greater aortic 18F-fluciclatide uptake than those with stable disease (TBR_max_ 1.29 vs 1.21, p=0.02).

**Conclusions:**

In vivo expression of α_v_β_3_ integrin in human aortic atheroma is associated with plaque burden and is increased in patients with recent myocardial infarction. Quantification of α_v_β_3_ integrin expression with 18F-fluciclatide PET has potential to assess plaque vulnerability and disease activity in atherosclerosis.

## Introduction

Atherosclerotic cardiovascular disease is the most common cause of death worldwide, and elucidating the mechanisms underlying the propagation and rupture of atherosclerotic plaques remains a key public health goal.^1^ Although our understanding of the pathogenesis underlying atherosclerosis has progressed over the last two decades, accurate prediction of clinical events remains elusive. There is therefore considerable interest in non-invasive imaging techniques that go beyond the detection of luminal stenoses and instead focus on measuring disease activity within the vasculature.^2 3^

Combined positron emission tomography (PET) and CT is a non-invasive hybrid imaging technique that integrates targeted functional molecular imaging with high-detail anatomical definition. This technique has been used to quantify vascular inflammation and calcification activity with success in both carotid and coronary atherosclerosis.^4–7^ Recently, intraplaque angiogenesis and neovascularisation has emerged as a key factor in the development, progression and instability of atherosclerotic plaques.^8^ The integrin alpha-V beta-3 (α_v_β_3_) cell surface receptor is upregulated on endothelial cells in states of angiogenesis and is also observed on macrophages at sites of increased vascular inflammation, another key contributor to plaque instability. This receptor helps coordinate interaction between cellular components and the extracellular matrix, and contains a distinctive RGD-amino acid sequence (the arginine-glycine-aspartate motif) in the cell-ligand interaction site. On this basis, several PET tracers targeting the RGD sequence have been developed for monitoring angiogenesis in malignant tumours.^9 10^ These tracers have also shown promise in monitoring atherosclerotic activity in preclinical models^8 11–14^ and in a recent small study of patients with carotid atheroma.^7^

18F-Fluciclatide is a novel RGD-based PET radiotracer with high affinity for the α_v_β_3_ integrin receptor.^10 15 16^ We hypothesised that 18F-fluciclatide may act as an imaging marker of atherosclerotic disease activity in vivo, informing about both inflammation and angiogenesis. In this study, we sought to characterise the cellular and imaging characteristics of 18F-fluciclatide uptake in human atherosclerosis using a clinical cohort of patients with both stable and unstable clinical disease.

## Methods

### Study populations

In total we studied 50 patients. Four patients were recruited who had sustained a recent stroke and were undergoing carotid endarterectomy. In these patients, excised carotid plaques were examined using histology and 18F-fluciclatide autoradiography. For in vivo imaging, 46 patients were recruited from the Royal Infirmary of Edinburgh between July 2013 and December 2014. This cohort comprised 19 *unstable* patients with a recent acute ST-segment elevation myocardial infarction (MI) (14±7 days after MI) (NCT01813045),^17^ and 27 *stable* patients with either stable angina (n=6) or asymptomatic atherosclerotic disease (n=21; 12 had calcific aortic valve disease) (NCT01837160). Exclusion criteria were age <40 years, women of childbearing potential, severe renal failure (estimated glomerular filtration rate <30 mL/min) or hepatic failure (Child-Pugh grade B or C), atrial fibrillation, known contrast allergy, inability to undergo scanning and inability to provide informed consent. All 46 subjects underwent 18F-fluciclatide PET imaging alongside clinical assessment that included evaluation of cardiovascular risk and high-sensitivity C-reactive protein (hs-CRP) measurement (Biocheck, Foster City, California, USA).

This study was conducted in accordance with the Declaration of Helsinki and with written informed consent of each participant.

### Radiosynthesis of 18F-fluciclatide

18F-Fluciclatide was manufactured at the Clinical Research Imaging Centre on an automated module (FASTlab synthesiser; GE Healthcare) by coupling an amino-oxy-functionalised peptide precursor (AH111695) with 4–18F-fluorobenzaldehyde at pH 3.5 to form 18F-fluciclatide.^17^ A full description of this synthesis has been published previously.^16^

### Histological validation

Carotid endarterectomy specimens (online supplementary data) were fresh frozen and sectioned in cryosection medium. Both atheromatous and non-atheromatous segments were fixed, stained with H&E and examined by immunohistochemistry for smooth muscle actin, CD31, CD68 and α_v_β_3_ integrin receptor expression before digital image capture (Axioscan .Z1, Zeiss, UK). Image analysis was performed on ImageJ32 software (National Institutes of Health, Bethesda, Maryland, USA). Staining was expressed as a percentage of the total plaque area and with an object size set threshold applied at 20×10 pixels, to limit counting to cell-sized objects. The density of cell staining in the endarterectomy tissue was expressed as cells per mm^2^.^18^

10.1136/heartjnl-2019-315103.supp1 Supplementary data

Autoradiography was performed to identify the precise localisation of 18F-fluciclatide binding in atherosclerotic tissue. Carotid sections were bathed in a solution of 18F-fluciclatide (1 kBq/mL) for 60 min and rinsed with phosphate buffer solution. To rule out non-specific radiotracer uptake, an un-labelled highly concentrated solution of fluciclatide was added to bind competitively with the α_v_β_3_ integrin receptors. 18F-Fluciclatide binding was imaged using a FujiFilm FLA-5100 Fluorescent Image Analyser (Raytek Scientific, Sheffield, UK).

### Clinical 18F-fluciclatide PET imaging

All patients in the imaging cohort underwent PET-CT imaging of the thorax with a hybrid scanner (Biograph mCT, Siemens Medical Systems, Erlangen, Germany) at the Clinical Research Imaging Centre, University of Edinburgh. Subjects were administered a target dose of 230 MBq 18F-fluciclatide. An attenuation correction CT scan (non-enhanced 120 kV and 50 mA, 3 mm slices) was performed prior to PET acquisition. To define tracer pharmacodynamics and the optimum timing of scanning, dynamic PET imaging of the thorax was performed in the initial 20 patients in three-dimensional (3-D) mode using a single bed position for 70 min. For the remainder of the study subjects, static imaging was performed at the optimal time point (found to be 40 min postinjection) using a single 30 min bed position in list mode with electrocardiographic gating. Immediately after PET acquisition, thoracic CT angiography was performed (online supplementary data).

### PET image reconstruction and analysis

Kinetic analysis was performed on the dynamic PET studies to investigate the pharmacodynamics of 18F-fluciclatide uptake within atheroma. The methodology is described in detail in the online supplementary data. In all patients, static electrocardiogram-gated PET images were reconstructed in diastole (40–70 min postinjection, 50%–75% of the R-R interval, Ultra-HD, 2 iterations, 21 subsets, zoom ×2, 200 pixels). Images were analysed by experienced observers blinded to the demographic data (WJ, AV) using an OsiriX workstation (OsiriX V.6.0 64-bit; OsiriX Imaging Software, Geneva, Switzerland). PET images were fused with the attenuation correction CT, and regions of interest drawn around the thoracic aorta on serial axial slices just beyond the discernible adventitial border. Aortic uptake was assessed in three regions: the ascending thoracic aorta (from the level of mid-right pulmonary artery up to the last slice where the aorta maintained its circular cross-sectional appearance), the descending thoracic aorta (the region extending from the tip of the diaphragm up to the last circular slice) and the aortic arch (the region of the aorta connecting the ascending and descending aorta). Within these regions, mean and maximum tracer activities were measured using standard uptake values (SUV; the decay corrected tissue concentration of the tracer divided by the injected dose per body weight, kBq/mL) and corrected for mean radiotracer blood pool activity to provide a mean of the TBR_max_ (mean TBR_max_).^5 19 20^ The blood pool radiotracer activity was quantified in the superior vena cava (SVC), measured in the axial plane on 4–5 sequential 5 mm axial slices above the level of the junction of the left innominate vein. In a substudy of 10 randomly selected subjects, images were assessed independently by two experienced observers and the interobserver reproducibility of 18F-fluciclatide SUV and TBR measurements assessed.

### CT image reconstruction and analysis

The aortic CT calcium score was calculated for the aorta as a whole and for its different regions using axial slices on the attenuation correction CT dataset (OsiriX V.6.0 64-bit; OsiriX Imaging Software) and expressed in arbitrary units (AU).^21^ In those patients whose descending aorta was visualised within contrast CT datasets (n=33), further measures of aortic atherosclerotic plaque burden were made on dedicated plaque analysis software (Vital Images, Minnetonka, Minnesota, USA). Using the sagittal plane, the entire portion of the descending thoracic aorta within the field of contrast CT acquisition was delineated and the luminal blood pool removed using semi-automated thresholding. The mean aortic wall thickness was recorded and corrected for the vessel diameter providing the indexed wall thickness. Additionally, the aortic wall volume was recorded and corrected for total vessel volume to provide an indexed plaque volume.

### Statistical analysis

Continuous data were tested for normality visually and with the D’Agostino-Pearson omnibus test. Continuous data were presented as median (IQR) and compared using Pearson’s or Spearman’s correlation or Wilcoxon signed-rank test as appropriate. Aortic calcium score and hs-CRP were log-transformed to base 10 to achieve normality prior to statistical analysis. Interobserver reproducibility was calculated by Bland Altman method and presented as mean bias ±2 SD, and intraclass correlation coefficients (ICC).^22^ Mann-Whitney U test was used to assess continuous variables across groups of a categorical variable. A matched stable atheroma group was created through censoring of patients with stable atheroma and high levels of aortic calcification by researchers blinded to the PET data. Statistical analysis was performed with GraphPad Prism V.6 (GraphPad Software, California, USA). A two-sided p<0.05 was taken as statistically significant.

## Results

### Plaque autoradiography and histology

**Figure 1:**
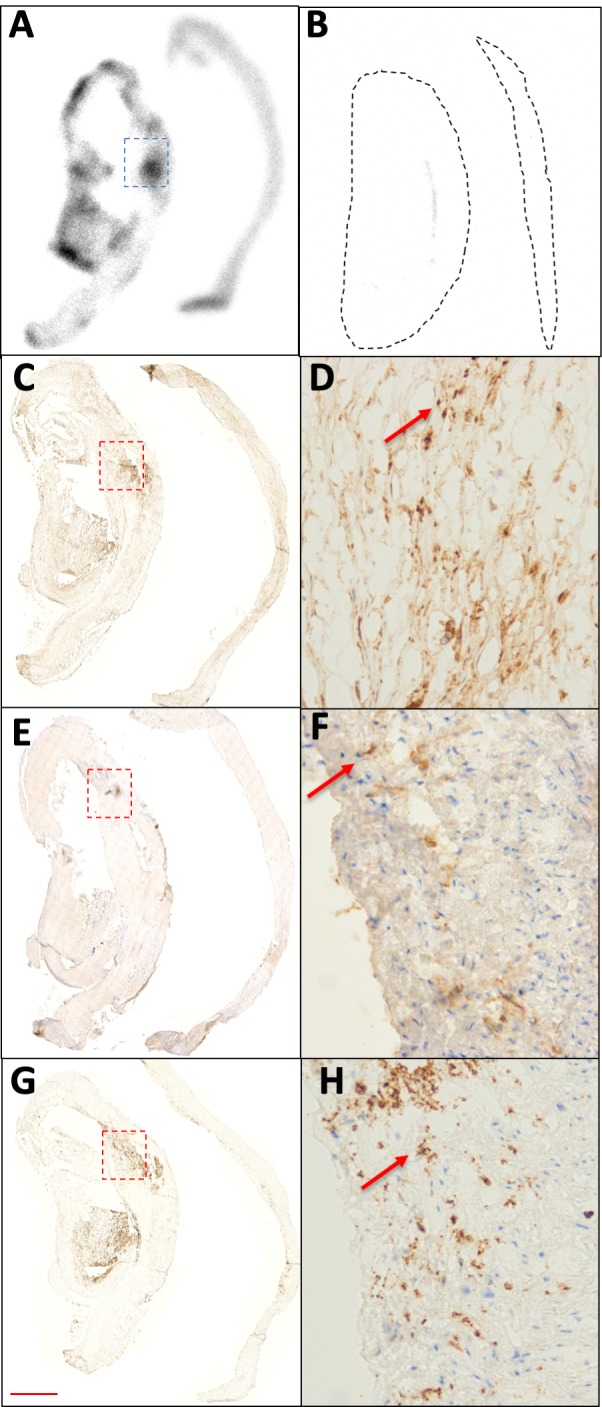
18F-Fluciclatide uptake in carotid atheroma. (A) Autoradiography image of segments of ruptured carotid plaque (left) and proximal healthy segments (right). Greater 18F-fluciclatide binding is visible within plaque rupture segments. Binding within the tissue segments (demarcated, black) was successfully blocked by the addition of a more concentrated un-labelled solution of fluciclatide (B). Areas showing the highest fluciclatide binding (demarcated, blue) exhibited a high degree of alpha-V beta-3 integrin expression (C and D, arrow) that also featured cellular staining for vascular endothelial cells (CD31, E and F, arrow) and inflammatory cells (CD68, G and H, arrow). Scale bar=1 mm in C, E, G and 75 µm in D, F, H.

Autoradiography of carotid plaque demonstrated focal 18F-fluciclatide binding in regions of advanced atherosclerosis that was effectively blocked by un-labelled fluciclatide (figure 1A, B). On adjacent tissue sections processed for histology, these regions of ex vivo 18F-fluciclatide uptake co-localised with areas of intense cellular staining of α_v_β_3_ integrin (45.2 (29.1–66.9) cells/mm^2^, % area 4.2 (3.2–5.7)), microvascular endothelial cells at sites of angiogenesis and positive remodelling (CD31+; 23.0 (6.5–63.0) cells/mm^2^, % area 2.5 (2.2–4.9)), and inflammatory macrophages deeper adjacent to the necrotic core (CD68+; 32.0 (21.0–49.8) cells/mm^2^, % area 3.7 (2.4–5.6), figure 1C–H). By comparison regions of carotid endarterectomy specimen without visible atheroma did not demonstrate 18F-fluciclatide uptake, and had much lower levels of α_v_β_3_ integrin receptor expression (4.5 (4.3–5.2) cells/mm^2^, % area 0.6 (0.5–2.0), both p=0.05 compared with areas with increased 18F-fluciclatide expression), as well as staining for both angiogenic endothelial cells (CD31+; 2.1 (1.8–2.4) cells/mm^2^, % area 0.9 (0.6–1.2), both p=0.05) and inflammatory macrophages (CD68+; 4.3 (2.8–4.4) cells/mm^2^, % area 0.6 (0.5–1.2), both p=0.03).

### In vivo imaging cohort

A total of 46 subjects underwent PET imaging with CT angiography (age 66±10 years, 74% male; table 1) following injection of 229 (IQR 217–237) mBq 18F-fluciclatide. Of the 46 subjects recruited, 19 had suffered a recent MI (*unstable cohort*), and 27 had imaging evidence of aortic atherosclerosis on CT but no recent cardiovascular events (*stable cohort*: 6 with stable angina and 21 with subclinical disease who were asymptomatic with no prior cardiovascular events). Interestingly, those in the stable cohort had greater aortic calcification than patients in the unstable group (AU (IQR); 326 (11–1114) vs 19 (0–483), p<0.01), reflecting their greater age (years (IQR); 69 (66–74) vs 60 (51–70), p=0.01). The groups were well matched for gender (p=0.97) and body mass index (p=0.39), and were both characterised by high prevalence of atherosclerotic risk factors, with more smokers in the unstable group (p<0.001) and more patients with hypertension in the stable group (p=0.02). No adverse events were reported following administration of 18F-fluciclatide and the average total radiation dose per participant was 15 mSv.

**Table 1:** Patient characteristics

	All (n=46)	Stable atherosclerosis (n=27)	Unstable atherosclerosis (n=19)	P value*
Age (years)	67 (60–74)	69 (66–74)	60 (51–70)	0.01
Male sex	34 (74)	20 (74)	14 (74)	0.97
BMI (kg/m^2^)	28 (25–31)	28 (24–30)	27 (25–32)	0.39
Systolic BP (mm Hg)	140 (124–160)	149 (134–167)	125 (114–139)	<0.001
18F-Fluciclatide dose (MBq)	229 (217–237)	228 (217–236)	229 (215–240)	0.69
Cardiovascular history				
Angiographically documented CAD	26 (57)	7 (26)	19 (100)	<0.001
Prev MI	24 (52)	5 (19)	19 (100)	<0.001
Prev PCI	20 (43)	2 (7)	18 (95)	<0.001
Prev CVD	4 (11)	4 (14)	0 (0)	0.08
Risk factors				
Current smoker	9 (20)	1 (4)	8 (42)	0.001
Diabetes mellitus	6 (13)	4 (14)	2 (10)	0.58
Prior hypertension	23 (50)	18 (67)	6 (32)	0.02
Prior hypercholesterolaemia	25 (54)	12 (44)	12 (63)	0.21
hs-CRP (mg/L)	3.5 (1.4–7.8)	2.7 (1.4–5.8)	5.6 (2.0–11.7)	0.08
Medications				
Aspirin	28 (61)	10 (37)	19 (100)	<0.001
Clopidogrel	19 (41)	4 (14)	19 (100)	<0.001
Statin	31 (67)	13 (48)	19 (100)	<0.001
β-Blocker	27 (59)	8 (30)	19 (100)	<0.001
ACEi/ARB	31 (67)	10 (37)	18 (95)	<0.001
Calcium channel blocker	7 (15)	6 (22)	1 (5)	0.11

Categorical data are displayed as n (%).

Continuous data are displayed as median (IQR).

*P values are quoted for comparisons between matched stable and unstable groups.

ACEi, ACE-inhibitor; ARB, angiotensin receptor blocker; AS, aortic stenosis; BMI, body mass index; BP, blood pressure; CAD, coronary artery disease; CVD, cerebrovascular disease; IHD, ischaemic heart disease; MI, myocardial infarction; PCI, percutaneous coronary intervention; hs-CRP, high-sensitivity C reactive protein.

### Dynamic analysis of aortic 18F-fluciclatide uptake

**Figure 2:**
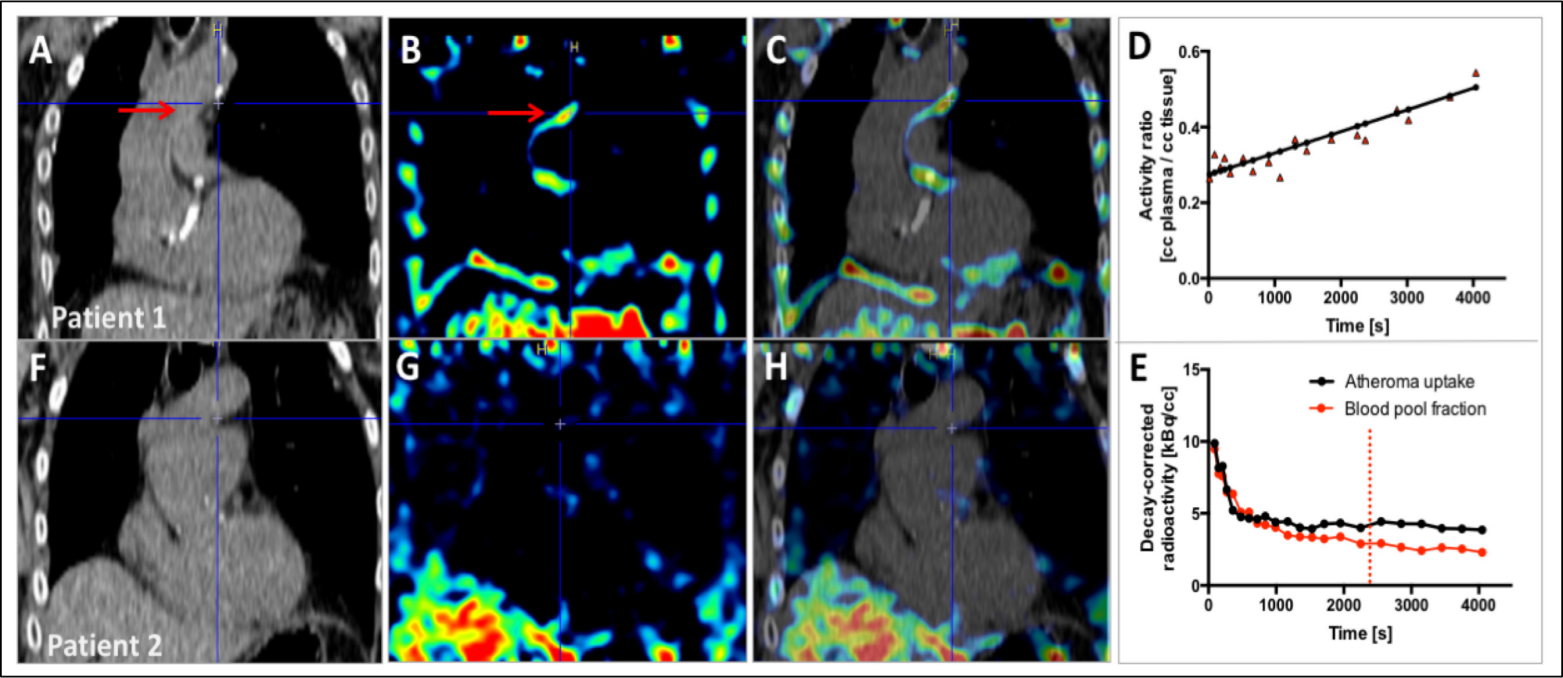
Kinetic analysis of aortic 18F-fluciclatide uptake. Sagittal views of the thorax following kinetic analysis in two participants with (patient 1: A–E) and without (patient 2: F–H) aortic arch 18F-fluciclatide uptake. CT images confirm presence (A) or absence (F) of aortic arch calcification as a marker of established atheroma. Patlak slope (Ki) parametric images (B and G) identify focal uptake within the aortic arch in the region of atheroma (red arrow) localising to the vessel wall on the fused Patlak and CT images (C and H). Patlak modelling (D) confirms irreversible integrin binding within the region of aortic arch calcification (red arrow). Time activity curves (TAC) within the same region (E) show a persistently high blood pool fraction, but uptake within atheroma that exceeds the blood pool fraction beyond 40 min (dashed line).

**Figure 3:**
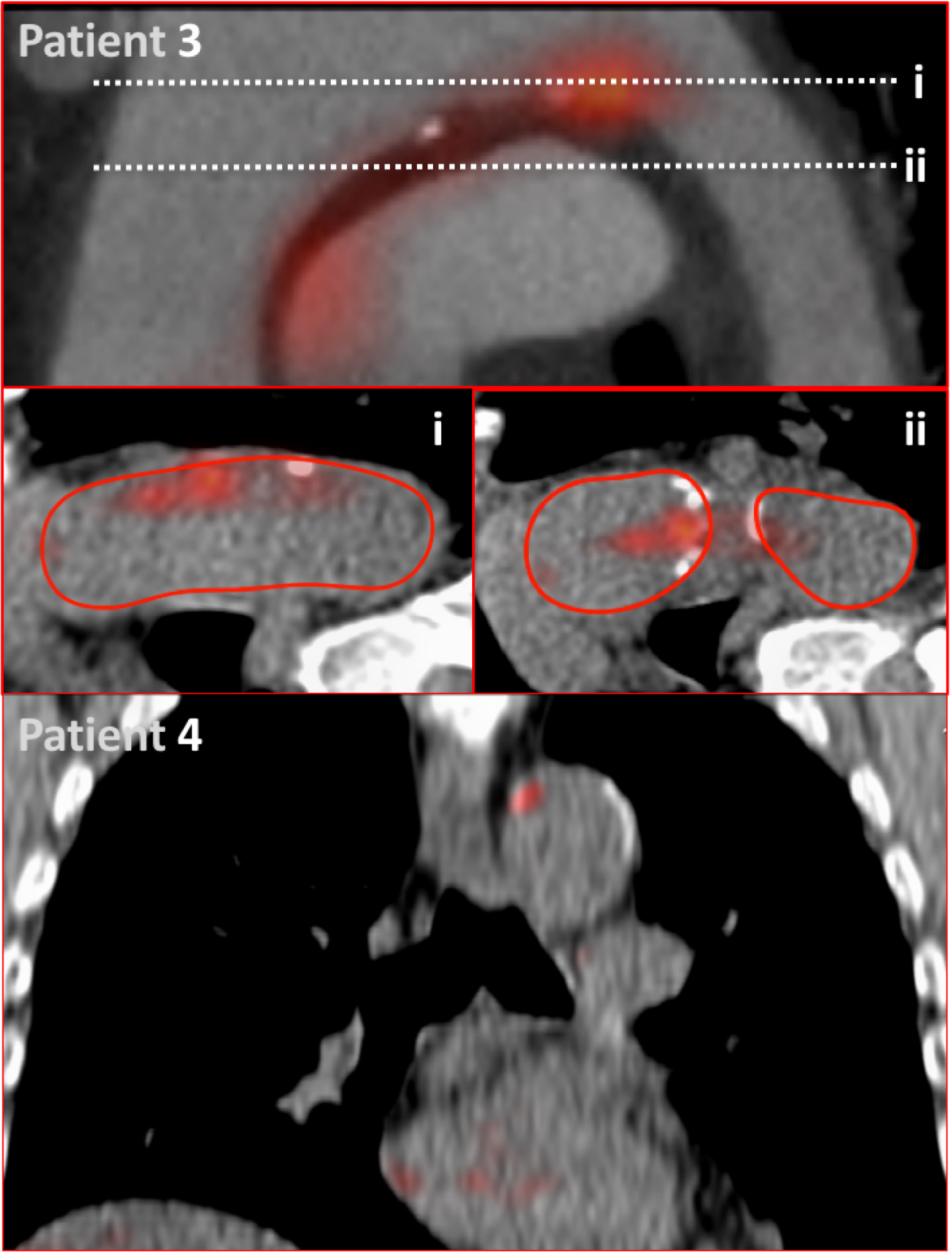
18F-Fluciclatide aortic uptake. Patient 3: modified coronal view of the aortic arch showing radiotracer uptake at the inner curvature of the aortic arch related to a region of aortic calcification. This activity is demonstrated on axial sections of the aorta (i and ii). Red lines indicate the adventitial borders of the aortic arch used for quantification of PET uptake. Patient 4: sagittal view of the thorax displaying focal 18F-fluciclatide uptake within a region of vascular calcification in the aortic arch.

On kinetic analysis (n=20), activity within aortic atheroma increased gradually, reaching a plateau at 40–70 min. Injected 18F-fluciclatide activity has a biexponential blood pool clearance with a half-life of about 10 min consisting of a fast redistribution component with a slower clearance component, causing relatively high residual blood pool activity during PET acquisition (40–70 min postinjection, SVC SUV_mean_ 2.73 (IQR 2.35–3.05); figure 2). Consequently, while 18F-fluciclatide uptake in aortic atheroma was measurable above background, TBR_max_ were relatively low (TBR_max_ range 1.08–1.68; tables 2 and 3, figures 2 and 3). In the 3-D Patlak slope (Ki) parametric images, six datasets (30%) were uninterpretable due to patient movement. In the remaining studies, increased aortic 18F-fluciclatide uptake was observed in half of subjects. Patlak modelling demonstrated a discernible linear phase suggesting irreversible ligand-receptor binding over the selected acquisition time and a greater Ki slope in these subjects compared with patients with no aortic uptake (figure 2). Subjects with uptake present on Patlak analysis (n=7) also appeared to have higher 18F-fluciclatide uptake on standard analysis (TBR_max_ 1.32±0.05 vs 1.19±0.03, p=0.08) and greater aortic calcification (aortic calcium score 704 (28–1788) vs 0 (0–119) AU, p=0.06) than those without visible uptake, although this did not reach statistical significance.

**Figure 4:**
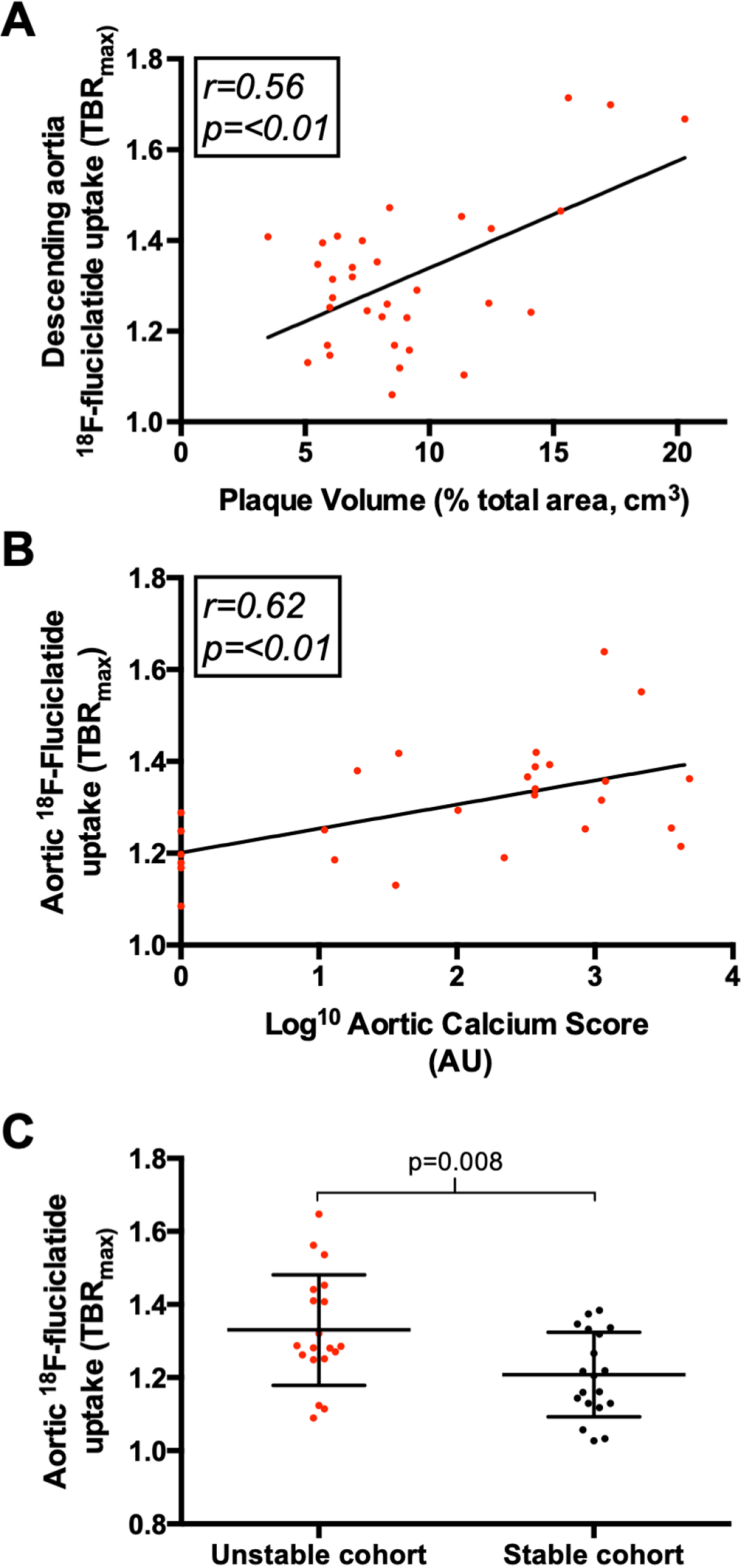
18F-Fluciclatide uptake, atheroma burden and clinical stability. Graphs displaying the relationship between aortic 18F-fluciclatide uptake and aortic plaque burden, assessed using both the plaque volume (A) and calcium score (B). Moreover, 18F-fluciclatide uptake was greater in patients with *unstable* (recent myocardial infarction) vs *stable* (no recent cardiovascular events) atherosclerotic disease (C). TBR, tissue-to-background ratio.

**Table 2:** Imaging results

	All (n=46)	Stable atherosclerosis (n=27)	Matched stable atherosclerosis (n=19)*	Unstable atherosclerosis (n=19)	P value†
18F-Fluciclatide PET uptake					
SVC (*SUV_mean_*)	2.73 (2.35–3.05)	2.66 (2.21–2.96)	2.47 (2.07–2.78)	2.88 (2.61–3.26)	0.01
Whole aorta (*mean SUV_max_*)	3.65 (3.04–4.01)	3.43 (2.95–3.81)	3.04 (2.86–3.61)	3.87 (3.46–4.12)	0.002
Whole aorta (*mean TBR_max_*)	1.31 (1.20–1.39)	1.29 (1.20–1.38)	1.25 (1.19–1.36)	1.32 (1.17–1.42)	0.32
Ascending aorta (*mean TBR_max_*)	1.32 (1.23–1.37)	1.27 (1.23–1.36)	1.24 (1.20–1.34)	1.33 (1.18–1.39)	0.10
Aortic Arch (*mean TBR_max_*)	1.28 (1.16–1.40)	1.27 (1.14–1.37)	1.21 (1.13–1.33)	1.29 (1.25–1.44)	0.02
Descending aorta (*mean TBR_max_*)	1.31 (1.19–1.42)	1.30 (1.23–1.41)	1.26 (1.17–1.41)	1.32 (1.12–1.45)	0.97
CT calcium score					
Whole aorta (*AU*)	95 (0–852)	326 (11–1114)	36 (0–469)	19 (0–483)	0.85
Ascending aorta (*AU*)	0 (0–11)	0 (0–46)	0 (0–0)	0 (0–0)	0.15
Aortic arch (*AU*)	29 (0–352)	102 (0–586)	13 (0–469)	0 (0–263)	0.76
Descending aorta (*AU*)	7.5 (0–78)	0 (0–123)	0 (0–123)	8 (0–71)	0.43
CTA plaque analysis (descending aorta)					
Mean wall thickness (*% vessel diameter*)	9.1 (6.5–12.8)	8.0 (6.3–10.0)	8.1 (6.3–9.8)	12.8 (8.6–18.4)	0.009
Plaque burden (*% total volume*)	8.3 (6.1–11.4)	7.4 (6.0–9.1)	7.5 (5.9–8.9)	11.4 (7.9–15.6)	0.008

Data are presented as median (IQR).

CTA, computed tomography angiography.

* Stable group subjects paired to equivalent calcium score in unstable group.

† P values are quoted for comparisons between matched stable and unstable groups.

AU, arbitrary units; CTA, Computed Tomography Angiography; PET, positron emission tomography; SUV, standard uptake value; SVC, superior vena cava; TBR, tissue-to-background ratio.

**Table 3:** PET uptake and baseline characteristics

	Total aortic 18F-fluciclatide uptake		
	All patients	Stable cohort	Unstable cohort
Continuous variables			
CT calcium score (log^10^ AU)	r=0.37 (0.08–0.60), p=0.01	r=0.62 (0.43–0.81), p<0.001	r=0.20 (−0.28–0.60), p=0.41
Mean wall thickness (% vessel diameter)*	r=0.57 (0.28–0.76), p<0.001	r=0.18 (−0.26–0.56), p=0.43	r=0.69 (0.15–0.91), p=0.02
Plaque volume (% total volume)*	r=0.56 (0.26–0.75), p<0.001	r=0.16 (−0.28–0.55), p=0.47	r=0.68 (0.13–0.90), p=0.02
Log_10_ hs-CRP (mg/L)	r=0.18 (−0.14–0.46), p=0.28	r=0.32 (−0.07–0.62), p=0.10	r=−0.24 (−0.63–0.24), p=0.06
Categorical variables			
Hypertension (mean TBR_max_)	1.33 (1.19–1.39) vs 1.29 (1.20–1.40), p=0.91	1.27 (1.21–1.38) vs 1.33 (1.19–1.38), p=0.90	
Established ischaemic heart disease (mean TBR_max_)	1.34 (1.22–1.42) vs 1.25 (1.19–1.35), p=0.15	1.37 (1.37–1.42) vs 1.25 (1.19–1.35), p=0.04	
Hypercholesterolaemia (mean TBR_max_)	1.34 (1.21–1.42) vs 1.27 (1.19–1.34), p=0.17	1.36 (1.29–1.41) vs 1.25 (1.19–1.30), p=0.01	
Diabetes mellitus (mean TBR_max_)	1.37 (0.32–0.45) vs 1.29 (1.19–1.38), p=0.09	1.37 (1.28–1.58) vs 1.29 (1.19–1.37), p=0.15	
Current smokers (mean TBR_max_)	1.32 (1.24–1.49) vs 1.29 (1.19–1.39), p=0.54	*N* too small	

*18F-Fluciclatide uptake assessed in the descending aorta only, to correspond with CT analysis.

AU, arbitrary units; PET, positron emission tomography; TBR, tissue-to-background ratio; hs-CRP, high sensitivity C-reactive protein.

### Aortic 18F-fluciclatide uptake reproducibility studies

Using static PET images, quantification of 18F-fluciclatide blood-pool activity demonstrated excellent interobserver reproducibility, with no fixed or proportional bias (SUV_mean_ mean difference; −0.11 (−0.36–0.15)) and a good ICC coefficient (0.95, online supplementary table S2). Quantification of aortic 18F-fluciclatide uptake using the established mean TBR_max_ approach^5 20^ also displayed no fixed or proportional bias (mean difference; 0.08 (−0.01–0.16)) with good ICC (0.92) (online supplementary table S1).

### Aortic 18F-fluciclatide uptake and atheroma burden

Our study cohort comprised patients with a wide spectrum of aortic atherosclerotic burden (aortic calcium score range 0–6857 AU). On static PET images, aortic 18F-fluciclatide uptake demonstrated a moderate relationship with disease burden in the aorta using both CT calcium scoring (r=0.37 (0.08–0.60), p=0.01) and the indexed plaque volume (r=0.56 (0.26–0.75); p<0.001) (table 3, figures 3 and 4). A good correlation was also observed with the relative vessel wall thickness (expressed as a percentage of vessel diameter, r=0.57 (0.28–0.76); p<0.001). There was no correlation between aortic 18F-fluciclatide uptake and the inflammatory marker CRP (log_10_ hs-CRP r=0.18, p=0.28).

### Aortic 18F-fluciclatide uptake in stable and unstable patients

To establish whether 18F-fluciclatide uptake may be a marker of unstable atherosclerotic activity, we compared aortic uptake in patients who had sustained a recent MI with those in our stable cohort. Given the association demonstrated between 18F-fluciclatide activity and plaque burden, we matched patients in the two groups according to CT calcium score (online supplementary table S2). We demonstrated increased 18F-fluciclatide aortic arch uptake in patients with recent MI compared with those in the stable cohort (TBR_max_; 1.29 (IQR 1.25–1.44) vs 1.20 (1.13–1.33, p=0.02)) despite equivalent aortic calcium scores (table 2, figure 4). Moreover, in patients with recent MI, a stronger correlation was observed between aortic plaque burden and 18F-fluciclatide uptake than in the patient population as a whole (aortic wall thickness r=0.68, p=0.02). When aortic SUV measurement were assessed, these were consistently increased in the unstable groups compared with the stable groups across all regions of the aorta, even before attempts were made to match these groups by calcium score (p<0.05 for all aortic regions, table 2 and online supplementary table S3).

### Aortic 18F-fluciclatide and cardiovascular risk factors

Among patients in the stable cohort, aortic 18F-fluciclatide uptake correlated with a number of risk factors (table 3). 18F-Fluciclatide activity was higher in patients with hypercholesterolaemia compared with those without (TBR_max_; 1.36 (IQR 1.29–1.41) vs 1.25 (IQR 1.19–1.30), p=0.01) and those with a diagnosis of coronary heart disease (TBR_max_; 1.37 (IQR 1.37–1.42) vs 1.25 (IQR 1.19–1.35), p=0.04) . An apparent observed trend was also seen in patients with diabetes (TBR_max_; 1.37 (1.28–1.58) vs 1.29 (1.19–1.37), p=0.15), although this did not meet statistical significance. Again, a strong correlation was observed between 18F-fluciclatide uptake and the CT calcium score (r=0.62, p<0.001) in this subgroup.

## Discussion

We present the largest multimodality imaging study to date evaluating the application of an RGD-based α_v_β_3_ integrin receptor radiotracer in the assessment of human atheroma. We have demonstrated that ex vivo 18F-fluciclatide binding co-localises to sites of α_v_β_3_ integrin receptor expression in excised carotid plaques, and this was associated with regions of both angiogenesis and inflammation. We have further demonstrated in vivo that 18F-fluciclatide uptake increased with progressive atherosclerotic plaque burden and is higher in patients with unstable versus stable atherosclerotic disease. These data would suggest that 18F-fluciclatide holds promise as a non-invasive marker of disease activity in atherosclerosis, informing us about two key characteristics of high-risk atheroma: inflammation and angiogenesis. RGD-based tracers may therefore aid our pathophysiological understanding of this important condition and help identify patients at increased risk of adverse cardiovascular events.

Plaque inflammation and angiogenesis are two key pathological processes associated with atheroma progression, plaque rupture and clinical events. Macrophages drive expansion of the necrotic core and secrete matrix metalloproteinases that weaken the fibrous cap, predisposing it to rupture. Angiogenesis is believed to occur in response to hypoxic conditions within the necrotic core and is associated with high-risk plaque characteristics.^3^ In addition, these new vessels are prone to leakage and rupture resulting in plaque haemorrhage that itself results in a pro-inflammatory response, plaque destabilisation and clinical events. A non-invasive imaging technique that can inform about the activity of these two adverse pathological processes might therefore be useful in identifying patients with active high-risk atheroma. Indeed, it is hoped that non-invasive markers of systemic atherosclerotic disease activity may identify the vulnerable patient and provide incremental risk prediction to an assessment of radiographic coronary or carotid disease burden alone.^2 23^ We have here demonstrated that 18F-fluciclatide PET-CT is an emerging and promising approach to achieve these aims.

Our autoradiographic data showed focal 18F-fluciclatide binding within carotid atheroma that localised histologically to α_v_β_3_ integrin expression with no evidence of non-specific binding. Regions of 18F-fluciclatide uptake on autoradiography also corresponded to sites of immunohistochemical cellular staining for vascular endothelial cells and macrophages. This is in keeping with previous RGD-radiotracer studies and supports its role as a selective marker for angiogenic and inflammatory components of atherosclerotic activity.^7 24 25^ Due to the close relationship between intraplaque inflammation and angiogenesis however, we are unable to ascertain whether 18F-fluciclatide was binding preferentially to one or the other of these processes.

Dynamic in vivo imaging studies in 20 subjects confirmed irreversible 18F-fluciclatide binding to regions of aortic atherosclerosis over the selected acquisition time, and demonstrated that the optimum time for 18F-fluciclatide imaging in the aorta is between 40 and 70 min. This timeframe was used for subsequent static PET imaging across the cohort as a whole. Static in vivo imaging demonstrated reproducible quantification of 18F-fluciclatide in aortic atheroma of all 46 patients, and this was correlated with the severity of aortic atherosclerotic burden, itself a manifestation of systemic atherosclerosis and a strong predictor of cardiovascular events.^26 27^ Indeed, associations were observed with the aortic calcium score, aortic wall thickness and overall plaque volume. In patients with clinically stable disease, aortic uptake of 18F-fluciclatide was increased in subjects with hypercholesterolaemia and those with ischaemic heart disease, with a trend towards higher uptake in patients with diabetes mellitus that did not meet statistical significance. Perhaps most importantly, 18F-fluciclatide uptake in aortic atheroma was increased in patients with unstable (recent MI) versus stable clinical disease. Interestingly, using 18F-fluorodeoxyglucose, we have previously demonstrated a similar pattern of increased metabolic activity in aortic atheroma among patients with recent MI.^28^ These data therefore lend support to the hypothesis that acute MI causes inflammation and instability in systemic atherosclerosis, as has been suggested in preclinical murine models.^29^ It also provides further validity to the concept that 18F-fluciclatide uptake can identify higher risk plaques. However, whether this increased disease activity truly represents a response to the infarct or rather the underlying trigger remains to be determined.

We acknowledge that there are limitations of our study that include potential partial volume artefacts, a limited histological assessment and the use of surrogate measures for aortic histology. 18F-Fluciclatide imaging prior to carotid endarterectomy would allow for a more direct comparison between in vivo imaging and histological assessment and consolidate our findings in line with previous publications.^7^ Furthermore, our exploratory assessment of aortic plaque volume may not take into account non-atheromatous intimal thickening in response to chronic hypertension. The dynamic imaging approach proved sensitive to patient movement during the prolonged acquisition period, limiting its utility in quantitative analysis, but this may ultimately be readdressed by novel motion tracking systems that allow for correction of cardiac and respiratory motion, enabling even greater definition of regional α_v_β_3_ integrin expression.^30^ Furthermore, given the limited number of patients studied using univariate analyses, we cannot exclude confounding of our results by other factors, nor that due to the multiple comparisons made, some tests may be statistically significant by chance alone. Nonetheless, we believe that the totality of our comprehensive evidence using multiple approaches and imaging modalities provides a robust and cogent argument to support our findings.

In conclusion, this is the largest study to date assessing α_v_β_3_ integrin expression in human atherosclerotic disease. We have demonstrated that 18F-fluciclatide uptake localises to regions of inflammation and angiogenesis, correlates with the plaque burden and is increased in patients with clinically unstable disease. Although further study is required, our data indicate that 18F-fluciclatide shows promise as a non-invasive marker of disease activity and instability in atherosclerosis.

**Key messages**

**What is already known on this subject?**

- The alpha-V beta-3 (α_v_β_3_) integrin receptor is a mediator of plaque angiogenesis and inflammation and has been targeted as a potential marker of atherosclerotic activity using positron emission tomography radiotracers.
- Studies of α_v_β_3_ integrin-specific radiotracers in humans are lacking.

**What might this study add?**

- This is the largest study to date assessing α_v_β_3_ integrin expression in human atherosclerosis.
- We have demonstrated that 18F-fluciclatide uptake localises to vascular inflammation and angiogenesis, correlates with plaque burden and is increased in patients with clinically unstable disease.

**How might this impact on clinical practice?**

- Non-invasive markers of atherosclerotic disease activity may identify vulnerable patients and provide incremental risk prediction to current anatomic imaging approaches.
- In this study, 18F-fluciclatide shows promise as a non-invasive marker of disease activity and instability in atherosclerosis.
