# Supplementary material for: In vivo alpha-V beta-3 integrin expression in human aortic atherosclerosis
Source: Heart. 2019 Aug 17;105(24):1868–75. doi: 10.1136/heartjnl-2019-315103 (PMC6929706; doi:10.1136/heartjnl-2019-315103)
Supplement: Supplementary data [file heartjnl-2019-315103supp001.pdf]

Supplementary Text and Data

Computed Tomography Acquisition

Immediately after PET acquisition, thoracic CT angiography was performed: 330 ms rotation time, 100 (body mass index <25 kg/m^2) or 120 (body mass index >25 kg/m^2) kV tube voltage, 160-245 mAs tube current, 3.8 mm/rotation table feed, prospective (heart rate regular and <60 /min), or retrospective (heart rate >60 /min) electrocardiogram-gated. Depending on body mass index, a bolus of 80-100 mL contrast (400 mgI/mL; Iomeron, Bracco, Milan, Italy) was injected intravenously at 5 mL/s, after determining the appropriate trigger delay with a test bolus of 20 mL contrast material.

PET Imaging

Kinetic Analysis

PET data were reconstructed (Ultra-HD, 2 iterations, 21 subsets, 256 pixels, 1.6-mm pixel size) in a dynamic profile using the following time frames; 60s x 5, 120s x 5, 180s x 5, 300s x 8. Regions of interest (ROI's) were drawn both in the blood pool and sites of aortic atheroma visible on CT and used to derive time activity curves after decay correction. These were used to define a timeframe for static imaging based upon the point at which optimum contrast between blood pool and tissue activity was observed. To define 18F-fluciclatide uptake in aortic atheroma, a kinetic modeling input function calculation was based on the PET image-derived activity curve from the aortic blood pool.(8) (PMod version 4.3.1, Pmod technologies limited, Switzerland). This input function was applied to a tissue activity curve generated from ROI's placed in the myocardial interventricular septum, to estimate the tissue influx rate *K*i (the slope

Jenkins WS, *et al. Heart* 2019; 105:1868–1875. doi: 10.1136/heartjnl-2019-315103

of the linear regression) and the volume of distribution (the intercept with the *y* axis) using a 2-tissue irreversible Patlak model, with t* set to 20 min, as described previously (9,11). Thoracic 18F-fluciclatide dynamic activity was then normalized for the blood-pool input function on a voxel-by-voxel basis, and after 3D Gaussian filtering (5-mm FWHM), a parametric 3-dimensional image of 18F-fluciclatide uptake was generated accordingly. Using this image, regions of 18F-fluciclatide binding in the vasculature were identified and manually delineated for subsequent *K*i analysis.

Histological Processing and Analysis

After obtaining informed consent, four human carotid intimal samples were obtained from patients undergoing carotid endarterectomy for symptomatic carotid artery atherosclerotic disease. Segments of dissected carotid atheroma were frozen in mounting medium. The tissue samples were then cut in sequential, longitudinal 4 µm and 20 µm slices sections at -20°C and thaw-mounted onto microscope slides. Effort was made to align segments of ruptured plaque alongside non-atheromatous segments within the same slide. The slides were then dried for 15 min and spray-fixed with neutral buffered formalin. After rinsing in distilled water the 4 µm sections were stained with hematoxylin-eosin (HE) and van-Gieson (VG) for conventional histopathological examination. In order to optimize immunohistochemistry, an antigen-unmasking step was performed by microwave treatment for 30 s. Endogenous peroxidase was blocked by incubation with hydrogen peroxide for 5 min. Sections were subsequently incubated with the primary antibodies; smooth muscle actin, CD31, CD68 (clone PG-M1), and integrin $\alpha_V\beta_3$

Jenkins WS, *et al. Heart* 2019; 105:1868–1875. doi: 10.1136/heartjnl-2019-315103

antibody, clone LM609 (Millipore) for 30 min at room temperature. After washing, the sections were incubated with Envision Flex (DAKO, K5007) for 30 min at room temperature, followed by incubation with diaminobenzamine (Sigma) for 10 min. The slides were finally counterstained with hematoxylin and digitally imaged (Axioscan.Z1, Zeiss, UK).

Clinical PET systems have limited resolution. To gain more detailed information about the precise localization of 18F-fluciclatide binding in atherosclerotic tissue, we undertook autoradiography. The 20 µm frozen sections adjacent to those used for immunohistochemical analysis were warmed to room-temperature and bathed in a solution of 18F-fluciclatide at a concentration close to in vivo imaging concentrations (1 kBq/mL) for 60 minutes and then rinsed with phosphate buffer solution. An unlabeled highly concentrated solution of fluciclatide was added to selected slides in order to competitively bind to $\alpha_v\beta_3$ to assess for non-specific tracer uptake. A freshly blanked phosphor screen was then placed over the slides and an overnight exposure undertaken. The screen was read using a FujiFilm FLA-5100 Fluorescent Image Analyser (Raytek Scientific Limited, Sheffield, UK). Sections were then manually registered and examined for co-localization with histological markers of atherosclerotic disease activity.

Jenkins WS, *et al. Heart* 2019; 105:1868–1875. doi: 10.1136/heartjnl-2019-315103

Table S1 – Reproducibility Analysis

18F-Fluciclatide Activity	Mean absolute difference[a]	Intra-class coefficient[b]
**Superior Vena Cava**		
**Mean SUV *(SUV [kBq/cc])***	-0.11 (-0.36 – 0.15)	0.947
**Aorta**		
**Mean SUV *(SUV [kBq/cc])***	-0.005 (-0.14 – 0.13)	0.986
**Mean SUV_{MDS} *(SUV [kBq/cc])***	0.01 (-0.17 – 0.15)	0.980
**Max SUV *(SUV [kBq/cc])***	0.07 (-0.13 – 0.27)	0.971
**Max SUV_{MDS} *(SUV [kBq/cc])***	0.06 (-0.22 – 0.34)	0.957
**Mean TBR**	0.04 (-0.02 – 0.10)	0.954
**Mean TBR_{MDS}**	0.04 (-0.04 – 0.10)	0.940
**Max TBR**	0.08 (-0.01 – 0.16)	0.912
**Max TBR_{MDS}**	0.07 (-0.03 – 0.17)	0.919
**$SUV_{Target\text{-}background}$**	0.19 (-0.05 – 0.43)	0.612

[a] Mean difference between TBR_{max} measurements (95% limits of agreement), and [b] ICC values for 18F-Fluciclatide throughout the thoracic aorta and SVC.

Abbreviations: ICC: intraclass correlation coefficient; MDS: most diseased segment; TBR: tissue to background ratio; SVC: Superior Vena Cava

Jenkins WS, *et al. Heart* 2019; 105:1868–1875. doi: 10.1136/heartjnl-2019-315103

Table S2 – Baseline demographic data

	**All** (n=46)	***Stable Atherosclerosis*** (n=27)	***Stable atherosclerosis; matched group*** *(n=19)*	***Unstable Atherosclerosis*** (n=19)	***P* value**
Age (years)	66±10	70±8	68±7	61±12	0.04
Male Sex	34 (74)	20 (74)	15 (79)	14 (74)	0.71
BMI (kg/m^2)	28±4	28±4	26±4	29±5	0.59
Systolic BP (mmHg)	140±22	149±19	151±18	127±19	<0.001
18F-Fluciclatide dose (MBq)	226±13	225±13	225±13	228±14	0.55
**Cardiovascular History**					
Angiographically documented CAD	26 (57)	7 (26)	1 (5)	19 (100)	<0.001
Prev MI	24 (52)	5 (19)	0 (0)	19 (100)	<0.001
Prev PCI	20 (43)	2 (7)	0 (0)	18 (95)	<0.001
Prev CVD	4 (11)	4 (14)	3 (16)	0 (0)	0.08
**Risk Factors**					
Current smoker	9 (20)	1 (4)	0 (0)	8 (42)	0.001
Diabetes Mellitus	6 (13)	4 (14)	2 (11)	2 (11)	0.99
Prior hypertension	23 (50)	18 (67)	11 (58)	6 (32)	0.11
Prior Hypercholester-olemia	25 (54)	12 (44)	6 (32)	12 (63)	0.03
hs-CRP (mg/l)	3.5 [1.4-7.8]	2.7 [1.4-5.8]	3.0±2.4	5.6 [2.0-11.7]	<0.001
Log_{10} hs-CRP (mg/l)	0.51±0.51	0.41±0.44	0.48±0.38	0.65±0.56	<0.001
**Medications**					
Aspirin	28 (61)	10 (37)	5 (26)	19 (100)	<0.001
Clopidogrel	19 (41)	4 (14)	3 (16)	19 (100)	<0.001
Statin	31 (67)	13 (48)	5 (26)	19 (100)	<0.001
β-Blocker	27 (59)	8 (30)	2 (11)	19 (100)	<0.001
ACEi/ARB	31 (67)	10 (37)	4 (21)	18 (95)	<0.001
Calcium Channel Blocker	7 (15)	6 (22)	4 (21)	1 (5)	0.11

Categorical data are displayed as n (%). Normally distributed data displayed as mean±SD. Non-normally distributed data displayed as median [interquartile range].

IHD - ischemic heart disease; AS - aortic stenosis; CAD - coronary artery disease; MI - myocardial infarction; CVD – cerebrovascular disease; PCI - percutaneous coronary intervention; hs-CRP - high sensitivity c-reactive protein; ACEi - ACE-inhibitor; ARB - Angiotensin Receptor Blocker.

[*] P-values are quoted for comparisons between matched stable and unstable groups.

Jenkins WS, *et al. Heart* 2019; 105:1868–1875. doi: 10.1136/heartjnl-2019-315103

Table S3 – Imaging Results

	All (n=46)	Stable Atherosclerosis (n=27)	Matched Stable Atherosclerosis (n=19) Ψ	Unstable Atherosclerosis (n=19)	*P* value*
**18F-Fluciclatide PET uptake**					
SVC (SUV_{mean})	2.74±0.49	2.62±0.46	2.54±0.40	2.9±0.48	0.02
Whole aorta (mean SUV_{max})	3.59±0.62	3.40±0.62	3.19±0.65	3.84±0.55	0.001
Ascending aorta (mean SUV_{max})	3.60±0.66	3.42±0.70	3.16±0.56	3.87±0.51	<0.001
Aortic Arch (mean SUV_{max})	3.51±0.62	3.29±0.61	3.05±0.52	3.84±0.48	<0.001
Descending aorta (mean SUV_{max})	3.61±0.68	3.46±0.65	3.28±0.65	3.83±0.66	0.01
Whole aorta (mean TBR_{max})	1.32±0.14	1.30±0.12	1.26±0.09	1.33±0.18	0.14
Ascending aorta (mean TBR_{max})	1.32±0.16	1.31±0.17	1.25±0.10	1.34±0.17	0.05
Aortic Arch (mean TBR_{max})	1.30±0.14	1.26±0.14	1.21±0.1	1.33±0.15	0.008
Descending aorta (mean TBR_{max})	1.32±0.17	1.32±0.13	1.29±0.11	1.33±0.21	0.56
**CT Calcium Score**					
Whole aorta (AU)	95 [0-852]	326 (11-1114)	36 (0-469)	19 [0-483)	0.85
Ascending aorta (AU)	0 [0-11]	0 (0-46)	0 (0-0)	0 [0-0}	0.15
Aortic arch (AU)	29 [0-352]	102 (0-586)	13 (0-469)	0 [0-263]	0.76
Descending aorta (AU)	7.5 [0-78]	0 (0-123)	0 (0-123)	8 [0-71]	0.43
**CTA Plaque analysis (descending aorta)**					
Mean wall thickness (% vessel diameter)	10.3±4.9	8.4±2.8	8.4±3.1	14.0±6.3	0.003
Plaque burden (% total volume)	9.1±3.9	7.7±2.3	7.7±2.5	12.0±4.7	0.004

Ψ *Stable group subjects paired to equivalent calcium score in unstable group.*

Jenkins WS, *et al. Heart* 2019; 105:1868–1875. doi: 10.1136/heartjnl-2019-315103

[*] P-values are quoted for comparisons between matched stable and unstable groups.

SUV - standard uptake value; SVC – superior vena cava; TBR - tissue-to-background ratio

Jenkins WS, *et al. Heart* 2019; 105:1868–1875. doi: 10.1136/heartjnl-2019-315103
